# Yersiniabactin-producing adherent-invasive *Escherichia coli* exploit host glycolysis to drive macrophage HIF-1α stabilization

**DOI:** 10.1126/sciadv.aeb7972

**Published:** 2026-07-23

**Authors:** Marlus S. Pedrosa, Ju-Hyun Ahn, John D. Sears, Kimberly A. Walker, Lance Thurlow, Nathaniel J. Moorman, Janelle C. Arthur

**Affiliations:** ^1^ Department of Microbiology and Immunology, University of North Carolina at Chapel Hill, Chapel Hill, NC 27599, USA.; ^2^ Department of Biomedical Sciences, Adams School of Dentistry, University of North Carolina at Chapel Hill, Chapel Hill, NC 27599, USA.; ^3^ Center for Gastrointestinal Biology and Disease, University of North Carolina at Chapel Hill, Chapel Hill, NC 27599, USA.; ^4^ Lineberger Comprehensive Cancer Center, University of North Carolina at Chapel Hill, Chapel Hill, NC 27599, USA.

## Abstract

The siderophore yersiniabactin (Ybt) produced by a subset of intestinal adherent-invasive *Escherichia coli* (AIEC) drive intestinal fibrosis in murine model of Crohn’s disease (CD). This is linked to the Ybt-induced disruption of host metal homeostasis and activation of the hypoxia-inducible factor 1–alpha (HIF-1α) in macrophages. Elevated glycolytic activity has been documented in both intestinal tissues and macrophages from patients with CD, indicating that metabolic reprogramming is a characteristic feature of the disease. Here, we show that HIF-1α stabilization by Ybt^+^ AIEC requires active host glycolysis. This effect is independent of *Hif1a* transcription and lipopolysaccharide stimulation and is not solely explained by intracellular bacterial load but instead relies on host metabolic activity. Mechanistically, Ybt^+^ AIEC activated the Akt-mTOR pathway to support HIF-1α translation. Inhibition of glycolysis suppressed this signaling axis, reducing HIF-1α translation and nuclear localization. Given the association between Ybt^+^ AIEC and fibrosis in CD, these findings suggest that targeting host glycolysis may limit AIEC-driven macrophage HIF-1α activation and fibrotic progression in CD patients.

## Teaser

A Crohn’s-associated gut bacterium exploits host glucose metabolism to induce profibrotic signaling in macrophages.

## INTRODUCTION

Inflammatory bowel diseases (IBDs) are chronic immune-driven inflammatory diseases affecting the gastrointestinal tract, often in the colon and ileum (*1*). The major types of IBD are ulcerative colitis (UC) and Crohn’s disease (CD). Patients with CD experience a high risk of developing intestinal fibrosis, a disease with no known cause, cure, or effective treatment (*2*). Intestinal fibrosis often leads to bowel obstruction and stricturing disease that is treated through surgical resection of the affected tissue (*2*). Although the mechanisms driving CD-associated fibrosis are poorly understood, they implicate intestinal microbiota. The CD-associated microbiota often includes an enrichment of Enterobacteriaceae, notably the subset of gut *Escherichia coli* termed adherent-invasive *E. coli* (AIEC) (*3*). These *E. coli* adhere to epithelial cells, invade macrophages, and persist and replicate within phagocytes (*3*, *4*). AIEC were first identified in CD lesions in 1998, and their association with patients with CD is well established (*5*, *6*). Their pathogenic potential in mouse models has been consistently demonstrated (*7*–*10*). Our laboratory has shown that small molecules produced by AIEC, such as colibactin and yersiniabactin (Ybt), can drive IBD-associated comorbidities, including colorectal cancer and intestinal fibrosis (*4*).

Metals are essential micronutrients required for survival across life forms, including resident bacteria and mammalian host cells in the intestines, and represent a major contributor to the maintenance of cellular homeostasis (*11*, *12*). Especially during inflammation, host cells start a robust “tug-of-war” to overcome the sequestration of essential metals to restrict the growth of invading microbes, a process known as “nutritional immunity” (*4*, *11*, *12*). One way microbes can evade nutritional immunity is by producing siderophores, high-affinity chelating compounds that enable microbial metal acquisition through specific uptake systems (*4*, *11*, *12*). *E. coli* produces several types of iron-binding siderophores, but among all, Ybt is unique because it can bind iron and other biologically important metals such as zinc (*13*–*15*). Our laboratory has demonstrated a previously unrecognized and critical role for Ybt beyond microbial metal acquisition: Once produced, Ybt can directly target the host and contribute to intestinal fibrosis (*7*, *16*). Recently, we demonstrated that Ybt sequesters zinc and activates hypoxia-inducible factor 1–alpha (HIF-1α) in macrophages, thereby contributing to CD-associated intestinal fibrosis (*16*).

HIF-1α is a master transcription factor that plays a central role in inflammatory and fibrotic diseases (*17*, *18*). Under normoxic conditions, HIF-1α is constitutively produced but rapidly hydroxylated by prolyl hydroxylase domain (PHD) proteins, targeting it for proteasomal degradation via the von Hippel-Lindau (VHL) pathway (*19*–*21*). However, under environmental insults such as low oxygen availability and infection, HIF-1α degradation is prevented, allowing it to translocate to the nucleus to exert transcriptional control of target genes (*18*, *22*). In addition to low oxygen and infection, multiple noncanonical inputs regulate the stabilization of HIF-1α by influencing its transcription or degradation (*23*). Metabolites such as succinate and lactate can inhibit PHD enzymes and promote HIF-1α stabilization, even under normoxic conditions (*24*–*29*). These noncanonical inputs collectively fine-tune HIF-1α regulation, which is critical for complex processes such as cellular metabolism and immune responses (*23*).

Cellular metabolism comprises an interconnected network of pathways that supply energy, redox equivalents, and biosynthetic intermediates to support cell function. Among these, glycolysis, the tricarboxylic acid (TCA) cycle, and oxidative phosphorylation (OxPHOS) form the core axis of energy production (*29*). Glycolysis breaks down glucose into pyruvate in the cytosol. In oxygen-rich conditions, pyruvate enters mitochondria and is further oxidized in the TCA cycle to fuel OxPHOS in the electron transport chain (*29*). However, under conditions of hypoxia, stress, or immune activation, cells often undergo metabolic reprogramming, favoring glycolysis over mitochondrial respiration (*29*, *30*). This metabolic reprogramming is especially prominent in immune cells such as macrophages, where these metabolic circuits are actively reshaped in response to infection to drive functional adaptation (*29*). This shift is not merely a passive adaptation but is actively controlled by other pathways, such as Akt–mechanistic target of rapamycin (mTOR) (Akt, also known as protein kinase B). The Akt-mTOR axis integrates environmental signals to coordinate metabolic activity with immune function (*31*). In macrophages, Akt-mTOR–driven metabolic and translational reprogramming is essential for mounting effective responses to pathogens (*32*). One critical target of the Akt-mTOR pathway is HIF-1α (*32*). The activation of the HIF-1α Akt–mTOR–HIF-1α pathway enables macrophages to generate adenosine 5′-triphosphate quickly and accumulate key metabolic intermediates that satisfy the energetic and biosynthetic demands of host-pathogen interactions (*25*, *29*, *33*). Thus, the Akt–mTOR–HIF-1α axis may function as a central signaling-metabolic hub that integrates cues from bacterial exposure with metabolic reprogramming and translational control. While much is known about the regulation of glycolysis by HIF-1α, how glycolytic activity influences HIF-1α stabilization in macrophages is largely unknown, especially in the context of host-pathogen interactions driving IBDs.

Recent studies have highlighted increased glycolytic activity in both intestinal tissues and macrophages from patients with CD, suggesting that metabolic reprogramming occurs during disease progression (*34*, *35*). Given the known links between glycolytic flux and HIF-1α stabilization targeting glycolysis, its role in regulating HIF-1α signaling may offer a promising avenue for therapeutic intervention in IBDs (*36*). Here, we demonstrate that AIEC-produced Ybt requires glycolytic support to sustain HIF-1α translation in macrophages. Our findings reveal that Ybt^+^ AIEC activate the Akt-mTOR pathway to promote HIF-1α protein synthesis, rather than prevent its degradation, linking bacterial virulence to host metabolic reprogramming. Inhibiting glycolysis suppresses Akt-mTOR signaling, impairs HIF-1α translation, reduces its nuclear accumulation, and limits intracellular bacterial load. This metabolic-translational axis not only reinforces the macrophage response to bacterial siderophores but may also contribute to the development of intestinal fibrosis in CD by promoting a profibrotic macrophage phenotype.

## RESULTS

### HIF-1α stabilization in macrophages requires metabolically active Ybt^+^ AIEC

**Fig. 1.**
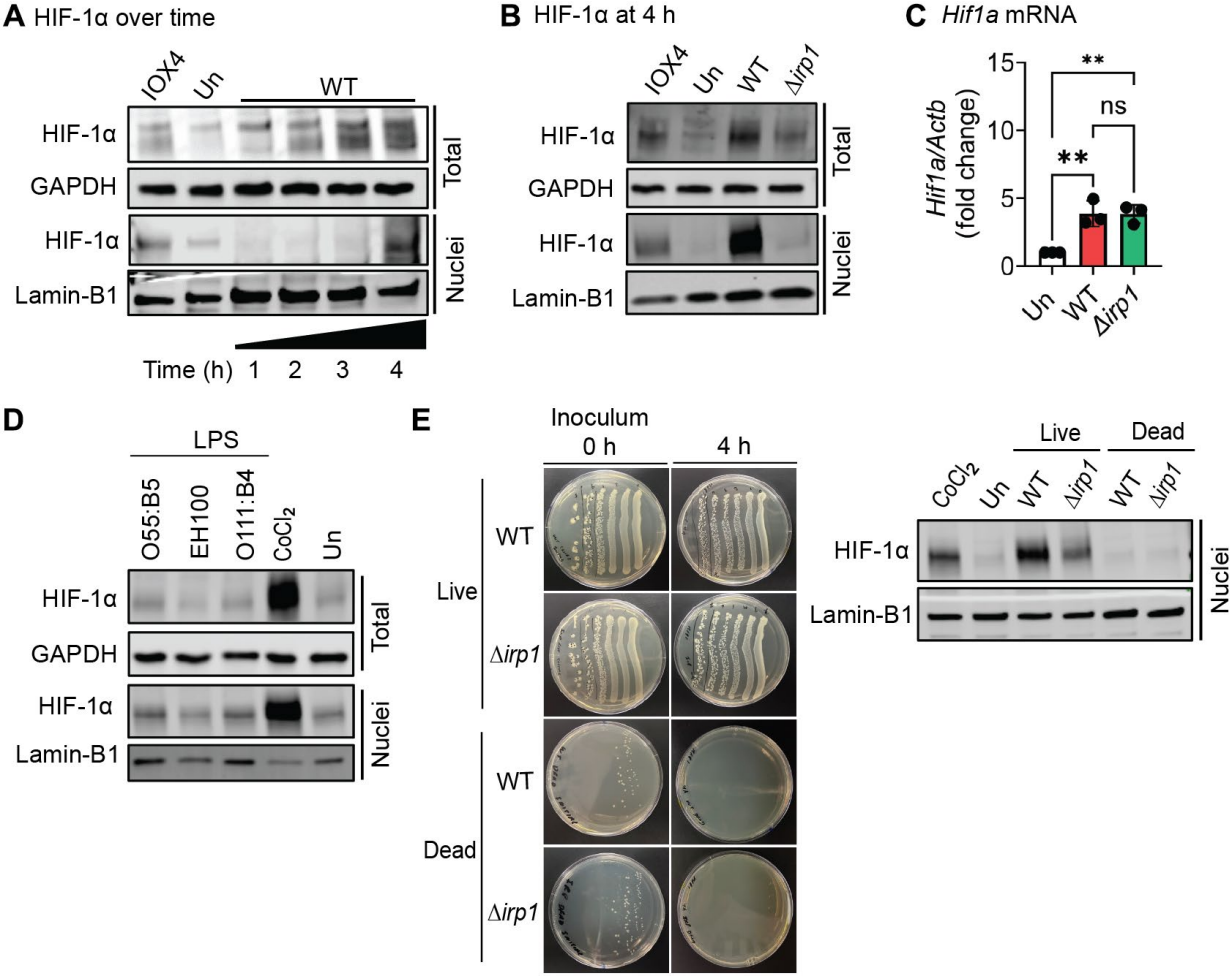
HIF-1α stabilization in macrophages requires metabolically active Ybt^+^ AIEC. (**A**) BMDMs were infected with Ybt-producing AIEC (WT) for up to 4 hours. Every hour, samples were collected for analysis. Total cell lysates and nuclear extracts were subjected to Western blot for HIF-1α. IOX4 (10 μM) was used as a HIF-1α–positive control. Glyceraldehyde-3-phosphate dehydrogenase (GAPDH) and Lamin-B1 were the loading controls for total cell lysates and nuclear extracts, respectively. (**B**) BMDMs were infected with WT and Ybt-deficient Δ*irp1* NC101 for 4 hours, and HIF-1α Western blots were performed as in (A). (**C**) BMDMs were infected as in (B), and the gene expression of *Hif1a* was measured by RT-qPCR. Fold change values were normalized to β-actin (*Actb*) and are presented relative to untreated (Un) cells. Data are presented as means ± SD (*n* = 3). Statistical significance was assessed using one-way ANOVA followed by Tukey’s post hoc test. (**D**) J774.A1 macrophages were treated with 1 μg/ml of different bacterial LPSs for 4 hours, and nuclear extracts were probed for HIF-1α as in (B). (**E**) J774.A1 macrophages were infected with live or gentamicin-killed strains. Image of culture plates confirm bacterial killing (left). HIF-1α was detected by Western blot of nuclear extracts as in (B). CoCl_2_ is a positive control. All blots shown are representative of three independent experiments performed with similar results.

We recently demonstrated that AIEC-produced Ybt stabilizes HIF-1α and promotes profibrotic macrophages (*16*). Here, we first confirmed the time course of HIF-1α stabilization upon infection of bone marrow–derived macrophage (BMDM) cells. We evaluated total and nuclear HIF-1α levels upon bacterial infection with Ybt-producing AIEC strain NC101 [wild type (WT)] and observed that HIF-1α protein was detectable starting from 2 hours in the total cell lysate and 4 hours in the nucleus (Fig. 1A). IOX4, a selective HIF prolyl-hydroxylase domain 2 (PHD2) inhibitor, was used as a positive control for HIF-1α stabilization (*37*). We next analyzed HIF-1α stabilization induced by infection with Ybt-producing (WT) and Ybt-deficient (Δ*irp1*) AIEC strain NC101 after 4 hours of infection (Fig. 1B). Ybt^+^ AIEC induced robust accumulation of nuclear HIF-1α in BMDMs compared to infection with the Ybt-deficient Δ*irp1*. Differences in HIF-1α protein stabilization are not attributed to differential gene expression of *Hif1a*, which is elevated ∼4-fold over untreated cells upon infection with either Ybt-producing or Ybt-deficient AIEC (Fig. 1C). As lipopolysaccharides (LPSs) can induce *Hif1a* transcription (*26*), we next determined the extent to which HIF-1α stabilization occurred in response to bacterial products using purified LPS and killed bacteria. We tested purified LPS from different *E. coli* serotypes (O111:B4, O55:B5, and EH100). None of these LPS induced HIF-1α stabilization greater than in untreated cells (Fig. 1D). In addition, we stimulated J774.A1 macrophages with gentamicin-killed bacteria (Fig. 1E, left) and evaluated HIF-1α protein levels (Fig. 1E, right), observing that bacteria must be alive to stabilize HIF-1α. The lack of HIF-1α stabilization by killed bacteria is not unexpected, as bacteria must be metabolically active to produce secreted small molecules, including Ybt. Together, we conclude that metabolically active Ybt-producing bacteria are required to induce macrophage HIF-1α stabilization, which cannot be attributed to differential *Hif1a* transcription or due to *E. coli* LPS alone.

### HIF-1α stabilization by Ybt^+^ AIEC can occur independently of intracellular uptake

**Fig. 2.**
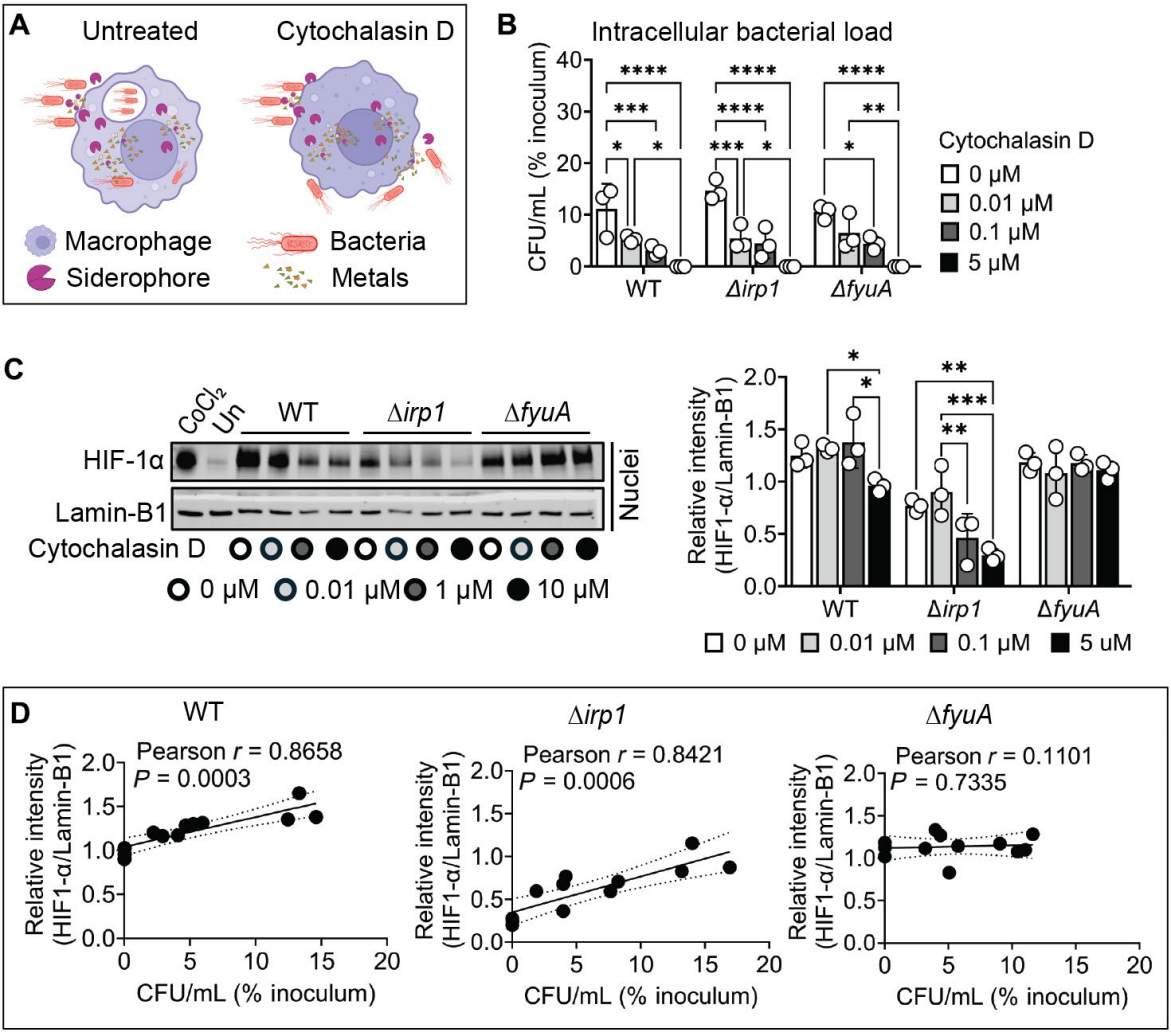
HIF-1α stabilization by Ybt^+^ AIEC can occur independently of intracellular uptake. J774.A1 macrophages were treated with increasing concentrations of Cyto D (0.01, 0.1, and 5 μM) and infected with Ybt-producing WT, Ybt-deficient Δ*irp1*, or Ybt-producing Δ*fyuA* strain NC101 for 4 hours (MOI 20). (**A**) Schematic representation showing that Cyto D prevents bacterial uptake. Created in BioRender. M. S. Pedrosa (2026); https://BioRender.com/nbxp0fr. (**B**) Bacterial internalization was evaluated by gentamicin protection assay. Data are presented as a percentage of inoculum, means ± SD. Statistical significance was assessed using one-way analysis of variance (ANOVA), followed by Tukey’s post hoc test. (**C**) HIF-1α was detected in nuclear extracts by Western blot (left). HIF-1α relative intensity was quantified using ImageJ software, measuring the HIF-1α band intensity and normalized to loading control Lamin-B1. The graph (right) shows these data as a fold relative to the assay control CoCl_2_ (*n* = 3). Data are presented as means ± SD. Statistical significance was assessed using two-way ANOVA, followed by Tukey’s post hoc test. (**D**) Pearson’s *r* correlation coefficients were calculated between intracellular CFU/ml (B) and normalized HIF-1α relative intensity (C). All blots shown are representative of three independent experiments performed with similar results.

AIEC are functionally defined by their ability to adhere/invade epithelial cells and survive/replicate within macrophages (*3*, *6*). Cytochalasin D (Cyto D) is a well-known drug used to inhibit phagocytosis and prevent the ability of bacteria to invade host cells (Fig. 2A). Our past results suggested that both extracellular and intracellular AIEC drive HIF-1α stabilization (*16*). To more directly test this, we exposed J774.A1 macrophages to increasing concentrations of Cyto D and infected them with Ybt-producing (WT and Δ*fyuA*) and Ybt-deficient Δ*irp1* AIEC strains. The Δ*fyuA* AIEC, which can produce Ybt but is unable to use it due to the deletion of the Ybt importer FyuA (*7*), induced consistently high HIF-1α levels even with undetectable bacterial internalization. In all strains, exposure to Cyto D decreased bacterial internalization in a dose-dependent manner (Fig. 2B). Even with undetectable bacteria levels observed with the highest drug concentration (5 μM), Ybt-producing bacteria strains stabilized HIF-1α to levels greater than untreated cells (Fig. 2C). HIF-1α levels correlated with intracellular bacterial load in macrophages infected with WT or Δ*irp1* strains, but not with Δ*fyuA* (Fig. 2D). Together, the data suggests that intracellular bacterial uptake is important for HIF-1α stabilization by Ybt-producing AIEC. However, Ybt^+^ AIEC that are unable to import Ybt (Δ*fyuA*) can stabilize macrophage HIF-1α from the extracellular space, without macrophage internalization. The Δ*fyuA* strain gives us a unique tool where internalization of Ybt^+^ AIEC is not required for HIF-1α stabilization.

### Glycolysis is required for HIF-1α stabilization by Ybt^+^ AIEC in macrophages

**Fig. 3.**
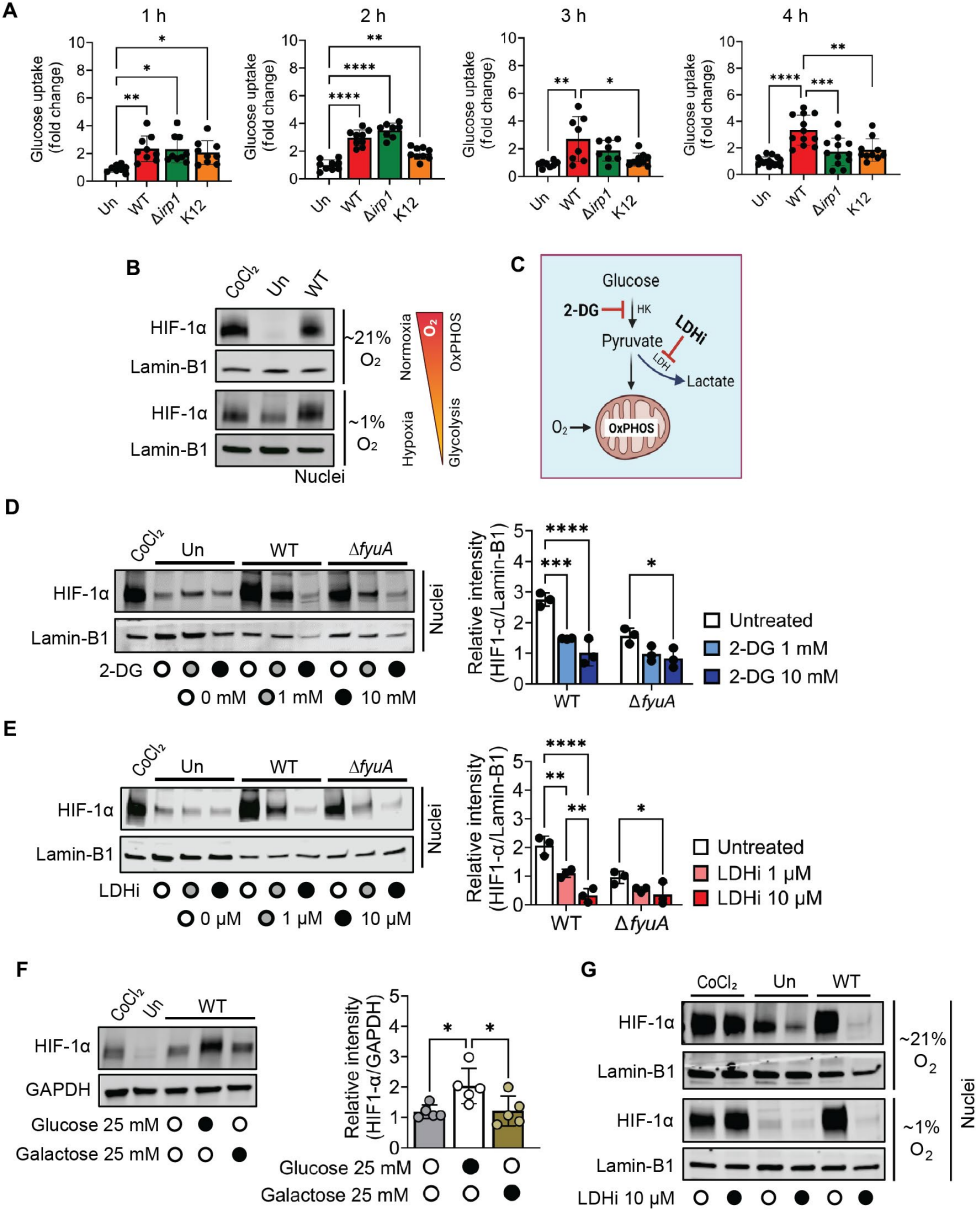
Glycolysis is required for HIF-1α stabilization and intracellular persistence of Ybt^+^ AIEC in macrophages. (**A**) Glucose uptake assay in BMDMs infected with Ybt-producing WT NC101, Ybt-deficient Δ*irp1* NC101, and Ybt-deficient *E. coli* K12. Data are presented as means ± SD. Statistical significance was determined by one-way ANOVA with Tukey’s post hoc test. (**B**) J774.A1 cells were infected with WT and Δ*irp1* NC101 in normoxic and hypoxic conditions for 4 hours. HIF-1α was detected by Western blot in nuclear extracts. CoCl_2_ is a positive control. Created in BioRender. M. S. Pedrosa (2026); https://BioRender.com/dk47lth. (**C**) Schematic representation of inhibitors used. Created in BioRender. M. S. Pedrosa (2026); https://BioRender.com/jq2zg5i. (**D** and **E**) J774.A1 cells were treated with (D) 2-DG (1, 10 mM) or (E) LDHi (1, 10 μM) and infected with WT or ΔfyuA NC101 for 4 hours. HIF-1α in nuclear extracts was analyzed by Western blot, quantified in ImageJ, and normalized to Lamin-B1(graph). Data represent means ± SD of three independent experiments (duplicates). Statistical significance was determined by two-way ANOVA with Tukey’s post hoc test. (**F**) BMDMs were infected with WT NC101 in glucose-free media supplemented with d-glucose or d-galactose (25 mM). HIF-1α in total lysates was quantified and normalized to GAPDH (graph). (**G**) J774.A1 cells were treated with LDHi (10 μM) and infected with WT NC101 under normoxia or hypoxia for 4 hours. HIF-1α was analyzed in nuclear extracts. Lamin-B1 and GAPDH served as loading controls for nuclear and total lysates, respectively. Blots are representative of three independent experiments. The circles indicate the presence (●) or absence (○) of the respective treatments indicated in the blots.

Metabolic changes in host cells are elicited upon encountering bacteria, with up-regulation of metabolic pathways such as glycolysis. HIF-1α plays a critical role in regulating both aerobic and anaerobic glycolysis (*33*). In addition, upon pathogen sensing, it is known that macrophages enhance glucose uptake to meet cellular demands (*33*, *38*). We observed that infection with AIEC and even the laboratory-adapted *E. coli* strain K12 exerted acute effects on glucose uptake over time (Fig. 3A). However, Ybt-producing AIEC sustain glucose uptake for a longer time, where at 4 hours postinfection, there is a significant elevation in glucose uptake induced by Ybt-producing WT versus the Ybt-deficient strains Δ*irp1* and non-AIEC K12. This timing is consistent with the elevated nuclear HIF-1α stabilization induced by Ybt-producing AIEC at 4 hours (Fig. 1B).

The colonic epithelium of the gut lumen is normally hypoxic, a condition referred to as “physiologic hypoxia” with a steep oxygen gradient between the gut lumen and submucosa (*39*). Hypoxia and HIF-1α signaling predominate normal intestinal metabolism and immune-mediated inflammatory conditions such as CD (*16*, *40*). We previously observed HIF-1α^+^ macrophages are abundant in human CD-resected strictures and in fibrotic lesions of IBD-susceptible mice (*16*). To determine whether Ybt-dependent HIF-1α stabilization depends on oxygen availability, we infected and subjected macrophages to hypoxia at 1% oxygen. We observed that Ybt-induced HIF-1α stabilization occurs in both normoxic and hypoxic conditions (Fig. 3B). Knowing that infection and low oxygen availability favor a metabolic shift in host macrophages from OxPHOS toward glycolysis (*29*, *30*), these results suggested that glycolytic support might be required for Ybt-induced HIF-1α stabilization. To test this hypothesis, we inhibited glycolysis using 2-deoxy-d-glucose (2-DG) and GSK2837808A (hereafter referred to as LDHi), which respectively target hexokinase II and lactate dehydrogenase A (Fig. 3C), two enzymes well-known as the first and last rate-limiting steps of glycolysis (*41*). Inhibiting glycolysis with either inhibitor, 2-DG (Fig. 3D) or LDHi (Fig. 3E), significantly reduced HIF-1α protein levels in a dose-dependent manner in J774.A1 macrophages. Similarly, primary BMDM treated with either glycolysis inhibitor exhibited significantly reduced HIF-1α protein levels (fig. S1A).

Functionally, the inhibition of glycolysis reduced intracellular bacterial survival (fig. S1B), which was not rescued by zinc supplementation (fig. S1C). The reduction in intracellular bacterial survival upon glycolytic inhibition may reflect altered host metabolic support for bacterial persistence, although this was not directly investigated in the present study. Under these glycolytic inhibition, HIF-1α levels positively correlated with intracellular bacterial (fig. S1D), indicating that reduced intracellular bacterial load likely contributes to the observed decrease in HIF-1α. However, this effect is not fully explained by differences in bacterial burden alone. Notably, HIF-1α levels were also reduced in macrophages infected with the Δ*fyuA* strain (Fig. 3, D and E), which can stabilize HIF-1α independently of bacterial uptake (Fig. 2), indicating that host glycolytic activity represents an additional requirement for HIF-1α stabilization. While the Δ*fyuA* strain uncouples bacterial uptake from HIF-1α stabilization under baseline conditions, this does not exclude a contribution of intracellular bacterial survival under glycolytic inhibition. Consistent with this, while HIF-1α levels are not correlated with intracellular bacterial load for the Δ*fyuA* strain under control conditions (Fig. 2), they positively correlate with intracellular bacterial load for both WT and Δ*fyuA* strains under glycolytic inhibition (fig. S1D). To exclude the hypothesis that these drugs could be bactericidal, we plated the supernatants to quantify extracellular bacteria at the same time point, 4 hours postinfection, which revealed no inhibition of extracellular bacterial growth (fig. S1E). We assessed bacterial growth over 24 hours in the absence of macrophages but the presence of the inhibitors and also observed no effect on bacterial growth (fig. S1F). These findings indicate that both intracellular bacterial survival and host metabolic activity contribute independently to HIF-1α stabilization.

Glucose is a preferred substrate for glycolysis; thus, another way to study the contribution of glycolysis is by using media devoid of glucose and supplemented with galactose. Galactose is metabolized more slowly than glucose as it must be first metabolized by the Leloir pathway before entering glycolysis (*42*). Accordingly, cells devoid of glucose or cultured in galactose-containing media rely more heavily on OxPHOS (*43*). To confirm our results, we infected BMDM with Ybt^+^ AIEC in culture media devoid of glucose and then supplemented with either glucose or galactose. Under these conditions, supplementation with glucose induced greater HIF-1α stabilization (Fig. 3F), as assessed in whole-cell lysates, compared to both glucose-free and galactose-substitute conditions, consistent with the higher glycolytic flux supported by glucose relative to galactose. The bacterial load was comparable across glucose, galactose, and glucose-free conditions, indicating that the differences in HIF-1α stabilization were not driven by differences in bacterial growth (fig. S1G). Notably, HIF-1α levels were not completely abolished, consistent with the acute nature of the metabolic perturbation in this model, in which cells were first recovered for ∼18 hours in glucose-containing medium to preserve viability and then subjected to short-term glucose withdrawal or substitution with galactose for the time of infection (4 hours), a time frame over which preexisting intracellular metabolites may continue to support residual glycolytic flux and HIF-1α stabilization.

In addition, as blocking glycolysis favors a metabolic shift toward OxPHOS, we verified the effects of LDHi on Ybt-dependent HIF-1α stabilization in hypoxia (Fig. 3G), a condition where OxPHOS is suppressed (*24*). We observed that inhibiting glycolysis with LDHi abolished Ybt-induced HIF-1α stabilization in both normoxia and hypoxia, suggesting that host cell glycolysis is required for Ybt^+^ AIEC to sustain macrophage HIF-1α stabilization.

### Glycolytic modulation of HIF-1α by Ybt^+^ AIEC is independent of *Hif1a* transcription

**Fig. 4.**
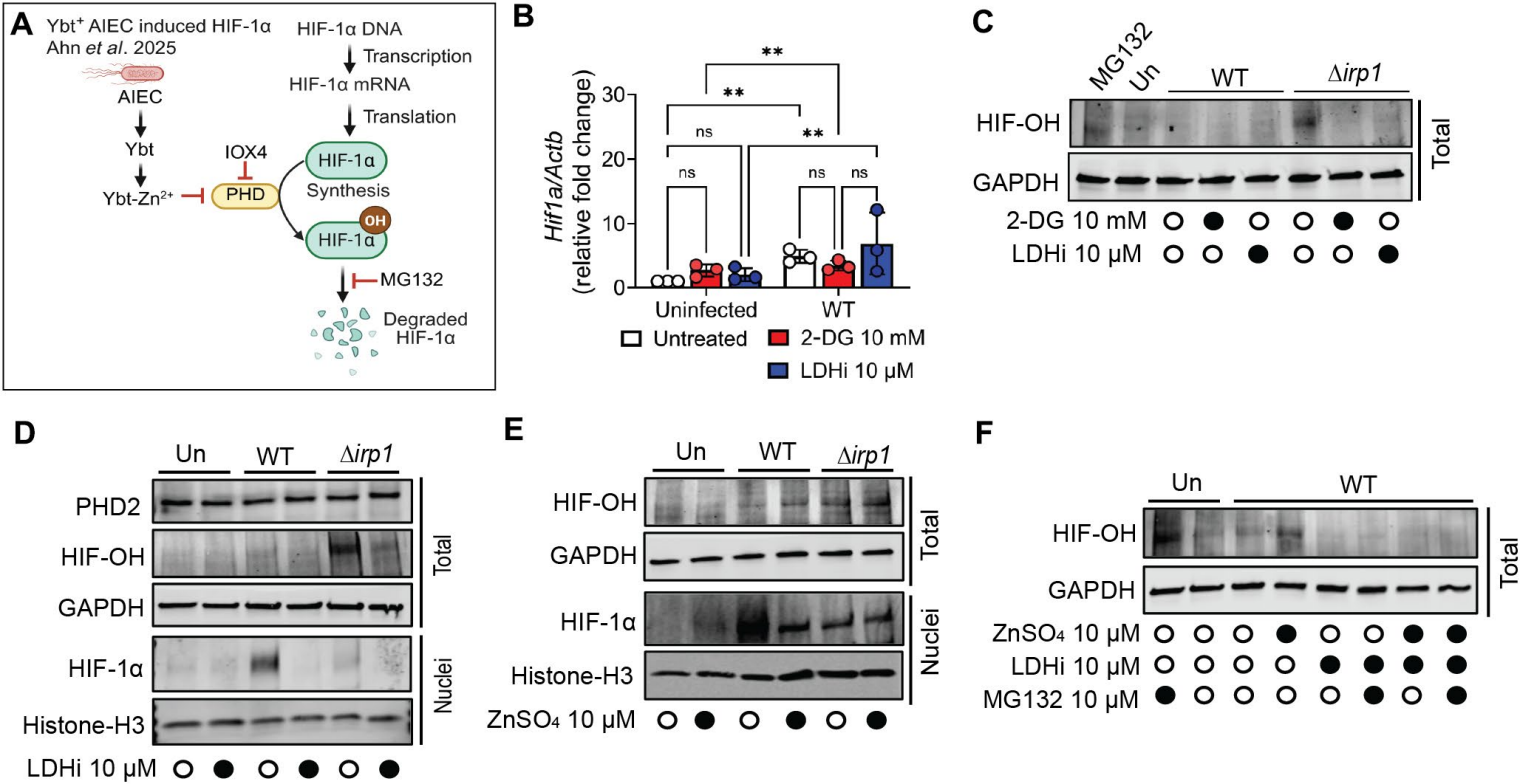
Glycolytic modulation of HIF-1α by Ybt^+^ AIEC is independent of transcription or canonical proteasomal degradation. (**A**) Schematic model of previous work: Ybt^+^ AIEC stabilize HIF-1α in macrophages through inhibition of PHD enzymes via Ybt-mediated Zn^2+^ sequestration. The model was used to test whether glycolysis regulates HIF-1α through transcription, translation, or degradation. IOX4 is a competitive inhibitor of PHDs, preventing hydroxylation of HIF-1α (HIF-OH); MG132 is a 26*S* proteasome inhibitor that blocks degradation of hydroxylated HIF-OH. Created in BioRender. M. S. Pedrosa (2026); https://BioRender.com/ggmj4al. (**B**) BMDMs were treated with 2-DG (10 mM) or LDHi (10 μM) and/or infected with WT NC101 (MOI 20) for 4 hours. *Hif-1*α mRNA was measured by RT-qPCR using 2^-ΔΔCt^ with *Actb* as the housekeeping gene, expressed as fold change relative to untreated cells. Data are presented as means ± SD from three independent experiments performed in duplicates. Statistical significance was assessed using two-way ANOVA, followed by Tukey’s post hoc test. (**C**) BMDM cells were treated as in (B) and/or infected with WT and Δ*irp1* NC101 for 4 hours. (**D**) BMDM cells were treated with LDHi (10 μM) and/or infected with WT and Δ*irp1* NC101 for 4 hours. (**E**) BMDM cells were treated with zinc (ZnSO_4_, 10 μM) and/or infected with WT and Δ*irp1* NC101 for 4 hours. (**F**) BMDMs cells were treated with zinc (ZnSO_4_, 10 μM), LDHi (10 μM), and MG132 (10 μM) and infected with NC101 for 4 hours. (C to F) Total cell lysates and nuclear extracts were used to quantify PHD2, HIF-OH, and HIF-1α by Western blot. GAPDH and histone-H3 were used as loading controls for total and nuclear extracts, respectively. All blots are representative of at least three independent experiments. The circles indicate the presence (●) or absence (○) of the respective treatments indicated in the blots. ns, not significant.

A variety of noncanonical signals contribute to the regulation of HIF-1α stabilization (*23*). These signals modulate HIF-1α levels by influencing both its transcriptional and posttranslational processes (*23*). Previously, we demonstrated that HIF-1α is stabilized due to the inhibition of PHD2 activity by Ybt^+^ AIEC (Fig. 4A), preventing HIF-1α hydroxylation (HIF-OH) and subsequent degradation (*16*). This mechanism is posttranslational and affects HIF-1α stability in a zinc-dependent manner. To determine whether glycolytic modulation affected HIF-1α at the levels of transcription, translation, hydroxylation, or degradation, we first examined the impact of 2-DG and LDHi on *Hif1a* mRNA levels and verified that these inhibitors did not affect *Hif1a* transcription (Fig. 4B). Metabolites such as lactate from glycolysis and succinate from TCA cycle stabilize HIF-1α by targeting the PHD enzymes (*26*, *27*, *44*, *45*). The disruption of glycolysis by 2-DG or by targeting LDH leads to a metabolic shift toward OxPHOS, where succinate pools are decreased due to its oxidation by the enzyme succinate dehydrogenase (SDH), thereby affecting HIF-1α stabilization (*26*, *44*). So, we hypothesized that the metabolic shift induced by the glycolytic inhibitors could be modulating lactate and succinate pools and consequently HIF-1α levels. We analyzed the effects of glycolytic inhibitors on LDH activity (fig. S2A) and lactate levels (fig. S2B) and observed that LDHi markedly affected LDH activity and lactate levels induced by AIEC. Next, we verified the effects of lactate and succinate on HIF-1α stabilization. In our model, lactate (fig. S2C) or succinate (fig. S2F) alone did not induce HIF-1α stabilization. In addition, we infected and treated cells with 2-DG and LDHi and supplemented the media with lactate (fig. S2D and S2E) or succinate (fig. S2, F and G). We observed that lactate or succinate add-back also failed to restore the HIF-1α stabilization abolished by these glycolytic inhibitors. In addition, we inhibited SDH using dimethyl malonate (DMM), a competitive and reversible inhibitor of SDH. By inhibiting SDH, DMM prevents the conversion of succinate to fumarate, resulting in the accumulation of succinate within the cell (*46*). DMM treatment did not restore HIF-1α levels under glycolytic inhibition (fig. S2H), as HIF-1α remained reduced relative to untreated conditions and comparable to LDHi treatment alone. Together, these data suggest that the modulation of Ybt^+^ AIEC-induced HIF-1α stabilization by glycolytic inhibitors is not explained by changes in transcription or alterations in succinate or lactate levels.

Among the several potential mechanisms of HIF-1α regulation, we considered the impact of glycolysis on HIF-1α protein degradation. Canonically, HIF-1α is hydroxylated by PHDs and targeted for proteasomal degradation (*25*, *29*, *33*). We first analyzed the impact of 2-DG and LDHi on HIF-OH levels and observed decreased Δ*irp1*-induced HIF-OH (Fig. 4C). We next examined the effect of LDHi on PHD2, HIF-OH, and nuclear HIF-1α (Fig. 4D). LDHi reduced HIF-OH levels without altering PHD2 abundance (Fig. 4D). Notably, nuclear HIF-1α levels are reduced under these conditions (Fig. 4D), indicating that the decrease in HIF-1α is not driven by enhanced hydroxylation and subsequent degradation. This lack of hydroxylation under LDHi treatment suggests that either HIF-1α is being targeted for degradation or is not being synthesized/translated and thus cannot be hydroxylated. We have shown that adding back zinc (Zn^2+^) rescues hydroxylation, leading to a reduction in nuclear stabilization of HIF-1α in macrophages infected with Ybt^+^ AIEC (Fig. 4E) (*16*). We hypothesized that if blocking glycolysis promotes HIF-1α degradation, then inhibiting the proteasome with MG132 and adding back zinc would abolish the effect of LDHi, leading to increased HIF-OH (Fig. 4A). We did not observe increased HIF-OH when proteasomal degradation was blocked and PHD activity rescued by zinc (Fig. 4F). These findings suggest the effects of glycolytic inhibition are not explained by increased hydroxylation or enhanced degradation and are distinct from the mechanism proposed for HIF-1α stabilization by lactate and succinate (*26*, *27*, *44*). Therefore, given that no changes were observed in HIF-1α transcription or its canonical degradation pathways, we propose that the ability of glycolysis inhibition to modulate HIF-1α stabilization by Ybt^+^ AIEC is likely attributable to reduced translation efficiency.

### Glycolysis supports Akt-mTOR–dependent translation of HIF-1α by Ybt^+^ AIEC

**Fig. 5.**
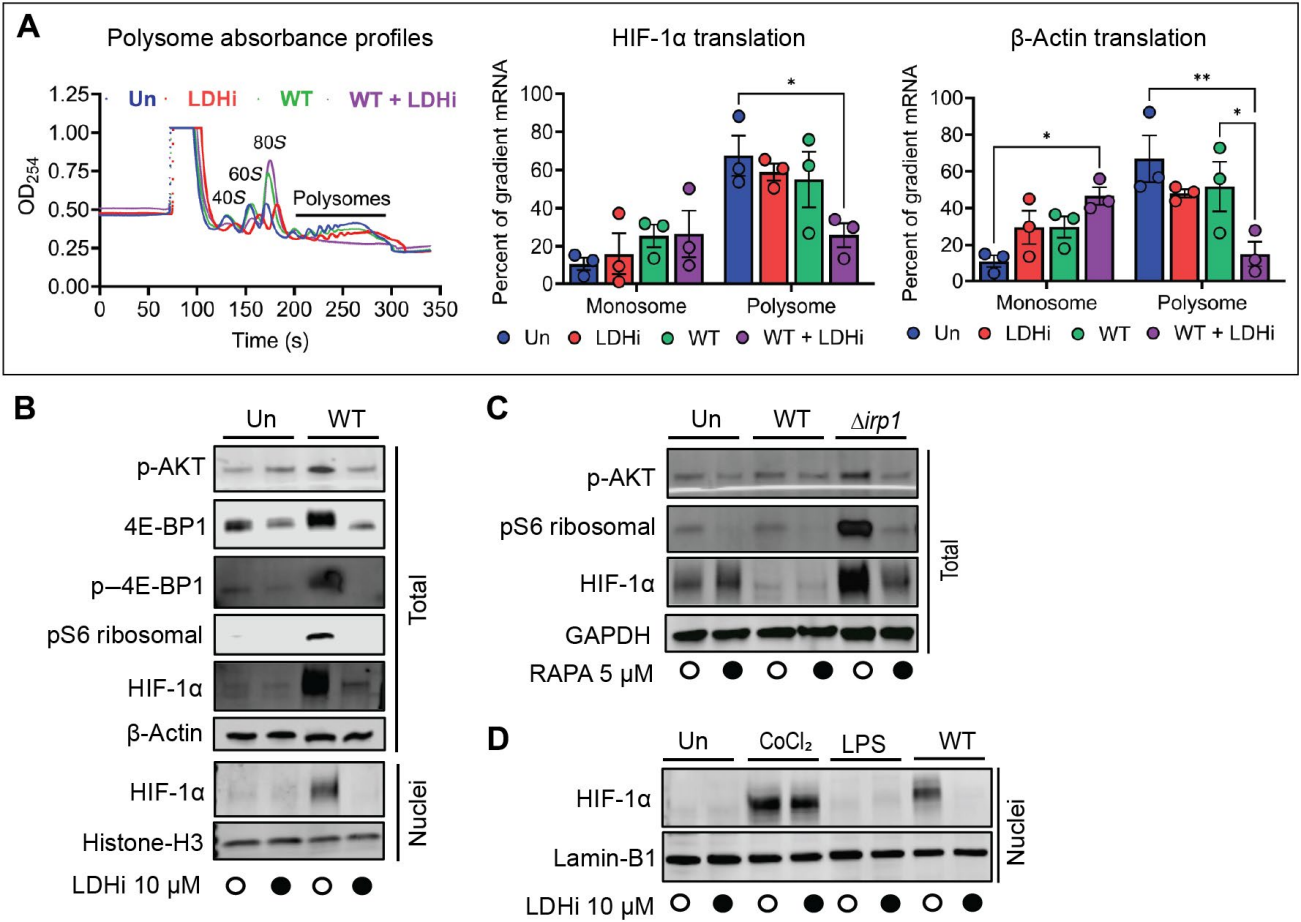
Glycolytic inhibition impairs HIF-1α synthesis by Ybt^+^ AIEC via Akt-mTOR. (**A**) Polysome profiling and translation efficiency analysis of HIF-1α and β-actin in J774.A1 macrophages under different conditions: untreated (Un), LDHi (10 μM), AIEC (WT), or AIEC WT + LDHi. Polysome absorbance profiles (left) were assessed by measuring OD_254_ across gradients. Translation efficiency of HIF-1α (middle) and β-actin (right) mRNAs was calculated as the ratio of transcript abundance in polysomes versus monosomes using RT-qPCR. (**B**). Immunoblot analysis of total and nuclear protein extracts from BMDMs infected with AIEC WT or left uninfected (Un) for 4 hours, with or without 10 μM LDH inhibitor (LDHi). Total lysates were probed for phospho-Akt, total 4E-BP1, phospho–4E-BP1, phospho-S6 ribosomal protein, HIF-1α, and β-actin (loading control). Nuclear extracts were probed for HIF-1α and Histone H3 (nuclear loading control). (**C**) Immunoblot analysis of total cell lysates from BMDMs treated with 300 μM CoCl_2_, infected with AIEC WT, or left untreated (Un), in the presence or absence of 5 μM rapamycin (RAPA) for 4 hours. Blots were probed for phospho-AKT, phospho-S6 ribosomal protein, HIF-1α, and GAPDH (loading control). (**D**) J774.A1 cells were infected with AIEC WT or treated with LPS (1 μg/ml; O55:B5) or 300 μM CoCl_2_, with or without 10 μM LDHi, for 4 hours. Nuclear extracts were analyzed by immunoblotting for HIF-1α and Lamin B1 (loading control). The circles below indicate the presence (●) or absence (○) of the respective treatments indicated in the blots. All graphs show means ± SD from three independent experiments performed in duplicates. All blots are representative of three independent experiments with similar results.

Using polysome profiling (*47*, *48*), we tested the hypothesis that glycolysis is required for efficient translation of HIF-1α in macrophages infected with Ybt-producing AIEC. This technique determines whether the disconnect between mRNA and protein levels of HIF-1α arises from regulation of translation independent of changes in mRNA levels. In this method, actively translated transcripts associated with multiple ribosomes (polysomes) are separated from those associated with preinitiated (40*S* and 60*S*) or single ribosomes (80*S*) that are grouped as monosomes. In general, an overall shift in RNA distribution from polysomes to monosomes suggests a global decrease in translation efficiency (*47*, *48*). Infection led to an increase in the 80*S* monosome peak, which when coupled with LDHi, also led to a corresponding decrease in polysome-associated mRNA (Fig. 5A, left). To determine whether this pattern applied specifically to transcripts of *Hif1a*, we extracted mRNA from across the gradient and performed quantitative reverse transcription polymerase chain reaction (RT-qPCR) on pooled monosome and polysome fractions. LDHi did not affect the *Hif1a* translation efficiency in uninfected macrophages (Fig. 5A, middle). However, LDHi decreased the HIF-1α translation efficiency in Ybt^+^ AIEC-infected macrophages (Fig. 5A, right), which is consistent with reduced levels of HIF-1α protein under these conditions. LDHi also decreased the translation efficiency of β-actin (Fig. 5A, left), indicating a reduction in global translation efficiency, rather than a selective effect on HIF-1α. However, we observed that only HIF-1α protein levels were reduced, while β-actin remained unaffected (Fig. 5B). Differences in protein stability and degradation rates can explain this discrepancy. HIF-1α has a short half-life of ∼5 min under normoxic conditions, as it is rapidly degraded (*49*). In contrast, β-actin has a significantly longer half-life (∼20 hours), making it much more stable and less susceptible to immediate changes in translation efficiency (*49*). While untreated J774.A1 cells already display high HIF-1α translation, likely due to their high glycolytic phenotype, this alone does not result in detectable protein accumulation due to active degradation. Notably, LDH inhibition does not reduce this baseline translational activity but selectively impairs HIF-1α accumulation during bacterial infection.

Glycolysis inhibition can impair both global and HIF-1α–specific translation primarily through mechanisms involving the Akt-mTOR pathway (*50*). To investigate the molecular mechanism underlying the translational suppression of HIF-1α by LDHi in the context of Ybt^+^ AIEC infection, we examined key components of the Akt-mTOR signaling pathway. In host macrophages, Ybt^+^ AIEC activated Akt, a kinase that functions upstream of mTOR (*51*, *52*). This activation was associated with increased phosphorylation of 4E-binding protein 1 (4E-BP1) (Fig. 5B), a translational repressor that, when phosphorylated by mTOR, releases eIF4E to allow cap-dependent translation (*52*). We observed increased phosphorylation of S6 ribosomal protein (Fig. 5B), a well-characterized downstream target of mTOR, which enhances ribosome biogenesis and global protein synthesis (*52*). These signaling changes coincided with elevated HIF-1α protein levels in both total and nuclear fractions (Fig. 5B), indicating enhanced translation and accumulation of this key transcription factor. LDHi blocked all these effects: Akt activation, 4E-BP1 and S6 phosphorylation, and HIF-1α stabilization. To further validate the involvement of mTOR signaling in HIF-1α translation during Ybt^+^ AIEC infection, macrophages were treated macrophages with rapamycin, a selective mTOR inhibitor (*53*). Western blot analysis revealed that rapamycin phenocopied the effects of LDHi, resulting in reduced phosphorylation of S6 ribosomal protein and decreased HIF-1α protein levels (Fig. 5C). The suppression of HIF-1α by rapamycin supports the conclusion that translation of HIF-1α is mTOR dependent in the context of Ybt^+^ AIEC infection. We treated J774.A1 macrophages with CoCl_2_, a PHD inhibitor and hypoxia mimetic, in the presence or absence of the LDH inhibitor, which did not diminish HIF-1α levels (Fig. 5D). These results indicate that glycolysis is dispensable for HIF-1α stabilization under classical hypoxia-mimetic conditions. This result, together with the polysome profiling data (Fig. 5A) and the observation that Ybt^+^ AIEC-induced HIF-1α stabilization occurs under hypoxic conditions (Fig. 3B), underscores a mechanistic distinction between classical hypoxia-induced and pathogen-driven HIF-1α stabilization. Our findings suggest that Ybt^+^ AIEC activates a pathogen-specific mechanism that requires intact glycolytic flux and is not replicated by hypoxia mimetics such as CoCl_2_ alone. These results reinforce our model in which host glycolysis and lactate metabolism sustain Ybt^+^ AIEC-induced HIF-1α stabilization through Akt-mTOR signaling. The pharmacologic inhibition of glycolysis with 2-DG and LDHi disrupts this metabolic signaling axis, thereby reducing both HIF-1α translation and nuclear accumulation. Collectively, these findings highlight the Akt–mTOR–HIF-1α pathway as a potential therapeutic target that may mediate the profibrotic effects of AIEC colonization in CD.

## DISCUSSION

Intestinal fibrosis can be a devastating complication of CD with no effective medical therapy (*2*). The interplay between microbial virulence factors and host cellular metabolism is increasingly recognized as a critical determinant of CD pathology (*54*). Macrophages are crucial to the gut immune system and in maintaining intestinal homeostasis (*53*, *55*). Alterations to their abundance and activity contribute to chronic inflammation, disrupted gut homeostasis, and the severity of intestinal inflammation and fibrosis (*4*, *16*). In this study, we provide mechanistic insight into how Ybt^+^ AIEC promote HIF-1α accumulation in macrophages and define a host metabolic axis critical for this response. While prior studies have emphasized transcriptional regulation and posttranslational stabilization of HIF-1α, particularly under hypoxic or inflammatory conditions, our data highlights a previously unrecognized mechanism by which Ybt^+^ AIEC subverts host macrophage glycolysis and lactate metabolism to promote HIF-1α stabilization.

HIF-1α is a central regulator of immune cell function that is activated in intestinal macrophages abundant in stricturing CD (*16*–*18*). We first confirmed and extended our earlier findings that HIF-1α is stabilized in macrophages infected with Ybt^+^ AIEC strain NC101 (Fig. 1, A and B) (*16*). This process occurs independently of changes in *Hif1a* transcription and is not replicated by stimulation with bacterial LPS or killed bacteria, indicating that metabolic activity and Ybt secretion are essential for the effect (Fig. 1, C to E). Moreover, using a Ybt^+^ strain lacking the Ybt importer FyuA, we show that phagocytosis is not strictly required for HIF-1α stabilization and that extracellular Ybt may be sufficient under certain conditions (Fig. 2, C and D). These findings suggest that secreted Ybt and its interaction with host cells drive HIF-1α stabilization. This finding is particularly intriguing given our previous work (*7*), which showed that colonization with the Ybt^+^ Δ*fyuA* strain results in greater fibrosis than the Ybt^+^ WT strain in mouse models. Although Ybt production from liquid cultures in vitro was comparable between Δ*fyuA* and WT strains (*7*), the inability of the Δ*fyuA* mutant to import Ybt may lead to greater extracellular accumulation in tissues and thus increased host exposure during infection. Our data suggest that Δ*fyuA* can stabilize HIF-1α even in the absence of significant bacterial internalization, likely due to reduced bacterial import and enhanced availability of Ybt to host cells. Thus, the ability of Δ*fyuA* to induce HIF1a even when excluded from the macrophage intracellular niche may contribute to its increased profibrotic potential.

While previous studies have attributed HIF-1α regulation primarily to oxygen-sensing mechanisms and proteasomal degradation (*23*, *55*), our findings suggest that HIF-1α stabilization by Ybt^+^ AIEC is mechanistically distinct from classical hypoxia-induced pathways. Enterobacteriaceae can modulate HIF-1α stabilization (*22*), supporting a growing interest in understanding how intestinal bacteria manipulate HIF-1α signaling, particularly in the context of chronic inflammatory diseases such as CD. Here, we build on these observations by showing that Ybt^+^ AIEC stabilizes HIF-1α through a multilayered mechanism that requires host glycolysis and lactate metabolism. Our findings identify a Ybt^+^ AIEC-dependent, oxygen-independent mode of HIF-1α activation that integrates host metabolic state with bacterial virulence. The pharmacological inhibition of glycolysis, either at the level of glucose uptake (using 2-DG) or lactate production (via LDHi), completely abrogated HIF-1α accumulation (Fig. 3, D and E) and significantly reduced intracellular bacterial persistence (fig. S1B). While glycolytic inhibition reduced intracellular bacterial survival, the effect on HIF-1α stabilization was not fully explained by bacterial burden alone, suggesting an additional requirement for host metabolic activity. Notably, glycolytic inhibition also reduced HIF-1α stabilization induced by the Δ*fyuA* strain, which can stabilize HIF-1α without detectable bacterial internalization, indicating that host metabolic activity contributes independently of bacterial uptake. In support, culturing macrophages in galactose-containing media, which forces a metabolic shift away from glycolysis, similarly suppressed HIF-1α accumulation (Fig. 3F). Together, these findings establish that host glucose and lactate metabolism are essential for HIF-1α stabilization by Ybt^+^ AIEC. While glycolytic inhibition also affects intracellular bacterial survival, these effects are not the primary focus of the present study and were not further investigated here.

Glycolysis and lactate metabolism support HIF-1α stabilization during Ybt^+^ AIEC infection by sustaining the metabolic and signaling conditions necessary for translation (Figs. 3 and 4). This mechanism distinguishes AIEC-induced HIF-1α accumulation from classical hypoxia-mediated stabilization (*23*). In our previous work, we showed that Ybt stabilizes HIF-1α by sequestering zinc, inhibiting PHD2 activity and thereby promoting HIF-1α accumulation (*16*). In contrast, here we found that blocking glycolysis with 2-DG or LDHi reduced HIF-1α protein levels without altering *Hif1a* mRNA (Fig. 4B) and that proteasome inhibition or zinc supplementation failed to restore HIF-1α under glycolytic inhibition (Fig. 4, E and F). In line with this interpretation, glycolytic inhibition reduces nuclear HIF-1α levels (Fig. 5B) without a corresponding increase in HIF-OH (Fig. 4, C and D), consistent with a mechanism not driven by enhanced hydroxylation. Instead, the inhibition of glycolysis reduces HIF-1α levels at a posttranscriptional level, acting upstream of zinc-dependent hydroxylation and proteasome-mediated degradation. These findings point toward impaired translation as the mechanism of HIF-1α loss when glycolysis is disrupted (Fig. 5A). We observed reduced polysome association of *Hif1a* mRNA and a corresponding decrease in HIF-1α protein levels, suggesting that upon inhibition of glycolysis, infection-induced *Hif1a* is not even translated into protein. The reduced translation efficiency of HIF-1α occurs in the context of decreased global protein synthesis, as evidenced by reduced β-actin translation. A reduction in global protein synthesis would have near immediate effects on the accumulation of proteins with short half-lives, including HIF-1α protein, estimated at ∼5 min under normoxic conditions. In contrast, proteins with a significantly longer half-life, including β-actin at ∼20 hours, would be less immediately affected at the level of steady-state protein accumulation despite reduced translation. Recently, a study in human embryonic kidney–293A cells found that glucose deprivation or 2-DG treatment reduces HIF-1α synthesis under hypoxia or dimethyloxalylglycine hypoxic mimetic (*50*). Puromycin labeling suggested less actively translated HIF-1α in parallel with reduced decreased global translation rates. The 2-DG did not reduce HIF-1α levels when HIF-1α simply accumulated by blocking its degradation using *VHL*^−/−^ cells (*50*), suggesting that the glycolysis inhibition primarily affects HIF-1α levels under conditions requiring active protein synthesis, such as infection or hypoxia. It is likely that differences in HIF-1α posttranscriptional control occur across cell types and in response to distinct stimuli. Our study provides a previously unknown insight into how host metabolic state influences HIF-1α protein synthesis in macrophages during infection.

These observations led us to investigate upstream signaling pathways that might link glycolysis to translation. We found that Ybt^+^ AIEC infection activates the Akt-mTOR axis, as evidenced by increased phosphorylation of Akt, 4E-BP1, and S6 ribosomal protein, classic markers of mTOR-mediated translational initiation (*25*, *51*, *52*, *56*) (Fig. 5B). This activation was abolished by LDHi, and critically, the inhibition of mTOR with rapamycin phenocopied LDHi, reducing both phosphorylation of downstream targets and HIF-1α accumulation (Fig. 5, C and D). These findings define a mechanism wherein host glycolysis and lactate metabolism maintain mTOR activity and together support the translational machinery required for HIF-1α synthesis during infection. In contrast, hypoxia mimetics such as CoCl_2_ stabilize HIF-1α protein via blocked degradation (*23*). In the context of Ybt^+^ AIEC infection, both the inhibition of degradation and activation of translation (via Akt-mTOR) are required for robust HIF-1α accumulation. By hijacking this pathway, Ybt^+^ AIEC enhance HIF-1α protein levels independently of transcription, leveraging host translational machinery downstream of Akt and mTOR. This distinct pathogenic strategy by Ybt^+^ AIEC fine-tunes the host metabolic and translational landscape to favor subtle but persistent changes in macrophage behavior. Thus, the effect is glucose dependent and aligns with broader evidence that immune cell activation is tightly coupled to metabolic reprogramming (*25*).

It is important to clarify that while Ybt^+^ AIEC infection activates mTOR, our data do not directly demonstrate that Ybt^+^ AIEC enhances the translation of HIF-1α above uninfected cells (Fig. 5A). This distinction is particularly relevant because the polysome experiments were conducted in J774.A1 macrophages, which already exhibit features of high glucose consumption and lactate production and showed elevated basal HIF-1α mRNA association with polysomes. In this metabolically reprogrammed background, Ybt^+^ AIEC may not further increase translation per se, but rather sustain or stabilize translation efficiency through mTOR activation, while tipping the balance toward HIF-1α protein accumulation via concurrent zinc sequestration by Ybt (*16*). Rather, we show that inhibition of host glycolysis using LDHi suppresses mTOR activity and downstream translational signaling, which is associated with reduced HIF-1α protein levels (Fig. 5B). This distinction is critical: Our findings support a model in which host glycolysis maintains the metabolic conditions necessary for Ybt^+^ AIEC-induced mTOR activation and subsequent HIF-1α accumulation, rather than Ybt^+^ AIEC directly promoting HIF-1α translation.

Aberrant activation of Akt has been associated with exacerbated intestinal inflammation and tissue damage (*57*). Moreover, mTOR signaling is activated in the intestinal epithelium of patients with active ulcerative colitis (*58*) and can promote inflammatory responses through the HIF-1α signaling (*59*). A study found that Ybt^+^ AIEC strain LF82 selectively activates epithelial mTOR signaling (*60*). In addition, evidence from other bacterial infections suggests that bacterial stimuli can trigger mTOR and HIF-1α signaling pathways (*61*, *62*). Although this prior work (*57*–*62*) has largely emphasized epithelial mTOR/HIF signaling, far less is known about whether AIEC engages the mTOR–HIF-1α axis in myeloid cells. Our data showing HIF-1α activation in macrophages in response to Ybt^+^ AIEC extend this pathway to an immune-cell context, supporting the idea that bacterial cues can modulate mTOR-HIF signaling across intestinal compartments. Meanwhile, several pharmacological agents targeting the mTOR pathway have shown therapeutic potential in patients with IBD (*63*, *64*). Other compounds that inhibit mTOR, such as celastrol (*65*) and docosahexaenoic acid (*66*), can reduce colitis severity in a preclinical mouse model of IBD. Therapeutically, this work provides a rationale for targeting the Akt–mTOR–HIF-1 axis in the context of CD, especially where Ybt^+^ AIEC colonization and HIF-1α accumulation are observed. mTOR inhibitors are already approved for use in other clinical contexts and may be candidates for repurposing. Furthermore, the pharmacologic inhibition of glycolysis may represent a novel strategy to disrupt host-microbe metabolic cross-talk and selectively dampen pathogenic signaling (*26*, *30*).

While our findings offer significant mechanistic insight, limitations remain. We used murine macrophage cultures (J774.A1 and BMDMs) to balance experimental rigor and physiological relevance. J774.A1 cells are an established and widely used system for quantitative bacterial uptake and intracellular survival assays, enabling reproducible mechanistic analyses of intracellular bacterial survival. J774.A1 cells were also used for experiments requiring high cell numbers and reproducibility, i.e. polysome analysis. Primary BMDMs were used to assess key findings in a system that more closely reflects metabolic regulation in vivo. The central findings were consistent across both macrophage models. These models do not fully replicate the intestinal microenvironment or account for human immune variability. Our in vitro model of infection is commonly used to precisely interrogate acute AIEC-macrophage interactions in a reproducible manner (*6*), but it does not fully reflect the complexity of the intestinal mucosa. In vivo, multiple cell types (including fibroblasts, epithelial cells, macrophages, and other various immune populations) interact within a dynamic and spatially organized microenvironment influenced by hypoxia, inflammatory cues, and a diverse microbiota. These contextual factors, as well as longer-term host–microbe interactions, are not captured in simplified monoculture or static models, which may limit the translatability of our findings.

We also focused on a single AIEC strain, NC101, which, despite being well-characterized and representative of Ybt-expressing CD isolates, may not encompass the full phenotypic variability observed across clinical AIEC strains. Differences in virulence traits, metabolic capacity, or host adaptability may influence the relevance and magnitude of the observed effects. Similarly, our analyses do not comprehensively assess the contribution of other HIF isoforms or nonmacrophage cell types that may also participate in intestinal inflammation and remodeling.

Moreover, although we observed reduced intracellular survival of AIEC under glycolytic inhibition, our study was not designed to establish long-term in vivo causality between host metabolic modulation, bacterial persistence, and fibrotic outcomes. Further in vivo studies are needed to define the full mechanism and its impact on long-term bacterial colonization and fibrosis in the inflamed intestines. Future work should assess how host-derived inflammatory or nutritional cues shape Ybt^+^ AIEC’s metabolic profile and whether these interactions vary across intestinal cell types beyond those studied here. Approaches such as metabolic tracing, organoid cocultures, and tissue-specific knockout models may provide additional resolution. Last, while targeting host metabolism appears promising, weather metabolic modulation can reverse established fibrosis or prevent progression at distint stages of disease remain an open and important question for future investigation.

In summary, these results reinforce the idea that Ybt^+^ AIEC induces a pathogen-specific, oxygen-independent HIF-1α stabilization program, which is finely tuned by host metabolic state. In this model, enhanced glycolysis and lactate metabolism are not only a metabolic signature of inflammation but also an active regulator of immune signaling, coordinating translational responses to Ybt^+^ AIEC infection. Targeting this axis, through inhibition of glucose and lactate metabolism and mTOR, may offer a promising strategy to selectively disrupt the metabolic dependency of Ybt^+^ AIEC-induced immune activation in CD disease.

## MATERIALS AND METHODS

### Bacterial strains and LPS

AIEC NC101 is a murine fecal isolate that produces Ybt and colibactin (*7*, *16*, *67*, *68*). Mutant strains that disrupt the production and/or utilization of Ybt (Δ*irp1* and Δ*fyuA*) were previously reported (*7*, *16*). Bacterial strains were preserved at −80°C and grown overnight at 37°C on Luria-Bertani (LB; Thermo Fisher Scientific, #BP9722-2) agar plates. Isolated colonies were transferred to M9 minimal-defined media supplemented with nicotinic acid (*68*) and grown overnight (∼15 hours) at 37°C with shaking at 220 rpm. Commercially available bacterial LPSs were obtained from *E. coli* strains O111:B4 (Sigma-Aldrich, catalog no. L3012), O55:B5 (Sigma-Aldrich, catalog no. L2637), and EH100 (Thermo Fisher Scientific, catalog no. NC1198692) and used at 1 μg/ml.

### Preparation of L929-conditioned medium

L929 fibroblasts were thawed from frozen aliquots (∼3 × 10^6^ cells/ml) and initially resuspended in 10 ml of Dulbecco’s modified Eagle’s medium (DMEM; Gibco, catalog no. 11995065) supplemented with 10% fetal bovine serum (FBS). Cells were seeded at 1 ml per T125 flask containing 50 ml of media and incubated at 37°C for 12 to 14 days without media change. At day 7, flasks were monitored to confirm cell health and cobblestone morphology. After incubation, the culture supernatant was harvested, centrifuged at 1000 rpm for 10 min to remove debris, and sterile filtered twice through 0.22-μm filters. Conditioned media were aliquoted and stored at −80°C for future use.

### BMDM cell isolation and infection

Bone marrow cells were isolated from femurs and tibias of WT C57BL/6 mice and infected as described previously (*16*). After a quick incubation in ammonium-chloride-potassium lysing buffer (Alkali Scientific, catalog no. DB0248) to remove red blood cells, the remaining bone marrow cells were differentiated in medium containing DMEM (Gibco, catalog no. 11995065), 20% heat-inactivated FBS, l-glutamine (Gibco, catalog no. 2503008), sodium pyruvate (Gibco, catalog no. 11360070), sodium bicarbonate (Gibco, catalog no. 2503008), and 30% L929 conditioned medium for 7 days on nontreated tissue culture plates (VWR International catalog no. NC1936598). After 7 days, cells were gently scraped and stored in liquid nitrogen or plated directly for experiments. BMDM were seeded in six-well plates (Corning, catalog no. 353060) at 1.5 × 10^6^ cells per well and allowed to adhere for 18 hours at 37°C and 5% CO_2_. Cells were infected at a multiplicity of infection (MOI) of 20 as determined by an optical density (OD_600)_ in serum-free media. Plates were spun at 1000 rpm for 5 min to settle bacteria into cells and then cocultured for up to 4 hours at 37°C and 5% CO_2_.

### J774.A1 macrophage infection

J774A.1 macrophage cells were purchased from the American Type Culture Collection and cultured in DMEM supplemented with heat-inactivated 10% (v/v) FBS. Bacterial infection was performed as previously described (*16*). In short, J774.A1 macrophage cells were plated at 3 × 10^5^ cells per well in six-well tissue culture plates and allowed to adhere and reach 70 to 80% confluency the next day (1 × 10^6^). Cells were then starved with 1% FBS medium for 18 hours and infected with bacterial strains in serum-free DMEM at an MOI of 20 as determined by OD_600_. In experiments measuring intracellular colony-forming unit (CFU) in relation to HIF-1α protein expression, duplicate plates were used to enable quantification of intracellular bacteria in parallel with generating cellular lysates for Western blots.

### Drug treatment

J774.A1 cells and BMDMs were seeded and treated in serum-free medium for 4 hours, following the infection protocol described above. All compounds were reconstituted according to the manufacturers’ instructions, and vehicle controls were included in all experiments. The following compounds were used: 2-DG (Thermo Fisher Scientific, catalog no. L07338.03), GSK2837808A (LDHi; Cayman Chemical, catalog no. 20626), d-glucose (Sigma-Aldrich, catalog no. G7021), d-galactose (Sigma-Aldrich, catalog no. 48260), Cyto D (Sigma, catalog no. C8273), rapamycin (MedChemExpress, catalog no. HY-10219), MG132 (MedChemExpress, catalog no. HY-13259), IOX4 (MedChemExpress, catalog no. HY-120110), zinc sulfate (Sigma-Aldrich, catalog no. Z0251), sodium lactate (Sigma-Aldrich, catalog no. 71716), succinic acid (Sigma-Aldrich, catalog no. 224731), DMM (Sigma, catalog no. 136441), and cycloheximide (CHX; Sigma-Aldrich, catalog no. C7698). Final concentrations and treatment conditions are provided in the corresponding figure legends.

### Hypoxia culture conditions

J774.A1 macrophages were cultured, infected, and treated as described above. Then, cells were placed in an oxygen-controlled incubator (Heracell VIOS 160i, Thermo Fisher Scientific, catalog no. 51033719). The oxygen concentration was precisely regulated to the desired oxygen concentration of 1% O_2_ balanced with nitrogen gas (N_2_) at 37°C with 5% CO_2_. The chamber atmosphere was continuously monitored using built-in sensors to ensure stability. Control samples were simultaneously incubated under normoxic conditions (∼21% O_2_, 5% CO_2_) in a standard tissue culture incubator.

### Gentamicin protection assay to measure bacterial uptake and survival

The intracellular survival of *E. coli* isolates within J774.A1 and BMDMs was assessed as previously described (*15*, *16*, *69*) using a standard AIEC assay with some modifications. After 4 hours of infection, the macrophage cells were washed two times with Dulbecco’s phosphate buffered saline (DPBS; Gibco, catalog no. 14190144), and fresh medium containing gentamicin (100 μg/ml) was added to kill extracellular bacteria. Following an additional 30 min of incubation, cells were washed two times with DPBS and lysed in 1 ml of 1% Triton X-100 in deionized water for 5 min. Bacteria were diluted and plated onto LB agar plates to determine the number of CFUs expressed as the mean percentage of bacteria present within cells at the time of collection compared to the inoculum (100%). To differentiate between the effects of culturing macrophages with live and dead bacteria, bacteria were killed with gentamicin as follows. Bacteria were cultured as described above, washed 2× with DPBS, and resuspended to an OD_600_ of 1 in DMEM (Gibco, catalog no. 11995065) with the addition of gentamicin (Gibco, catalog no. 15750060) at a final concentration of 100 μg/ml, which kills extracellular bacteria, and incubated for 1 hour. J774.A1 macrophages were infected with live or gentamicin-killed strains for 4 hours. Before infection (T0: live and dead inoculum) and after 4 hours, bacteria were diluted and plated onto LB agar plates to determine the CFU and confirm bacterial killing.

### Immunoblotting

Total cells were lysed directly by 2× Laemmli reducing buffer. Nuclear and cytoplasmic proteins were isolated from macrophages using the NE-PER Nuclear and Cytoplasmic Extraction Reagents Kit (Thermo Fisher Scientific, catalog no.78833) according to the manufacturer’s protocol. Protein quantification was performed using a Qubit 4 Fluorometer (Thermo Fisher Scientific). Immunoblotting was performed as previously reported (*16*) using the following primary antibodies: HIF-1α (Novus Biologicals, catalog no. NB100-479), Lamin B1 (Santa Cruz Biotechnology, catalog no. SC374015), phospho-Akt (Ser^473^; Cell Signaling Technology, catalog no. 9271T), Akt (Cell Signaling Technology, catalog no. 9272), β-actin (Invitrogen, catalog no. MA5-15739-D680), glyceraldehyde-3-phosphate dehydrogenase (Invitrogen, catalog no. MA515738D68), 4E-BP1 (Cell Signaling Technology, catalog no. 9452S), phospho-S6 ribosomal protein (Ser^235/236^) (Cell Signaling Technology, catalog no. 2211), and phospho–4E-BP1 (Thr^37/46^; Cell Signaling Technology, catalog no. 2855S). Total lysates (10 μg) or nuclear extracts (5 μg) were resolved by SDS–polyacrylamide gel electrophoresis and transferred to Immobilon-FL polyvinylidene difluoride membranes. Membranes were blocked in TBST (TBS with 0.1% Tween 20) containing 5% nonfat milk for 1 hour at room temperature, followed by overnight incubation at 4°C with primary antibodies diluted in 3% bovine serum albumin in TBST. After washing, membranes were incubated with appropriate secondary antibodies for 1 hour at room temperature. Protein bands were visualized using an Odyssey DLx Imager (LI-COR Biosciences). HIF-1α relative intensity was quantified using ImageJ software, normalized to the loading control, and expressed as a fold relative to the positive control CoCl_2_. All immunoblots are representative of at least three independent experiments.

### Reverse Transcription Quantitative PCR (RT-qPCR)

According to the manufacturer’s instructions, total RNA was extracted from cells grown in six-well plates using RNeasy Mini kit (QIAGEN, catalog no. 74106). The concentration and purity of the RNA were assessed by measuring absorbances at 260 and 280 nm using the NanoDrop (Thermo Fisher Scientific). cDNA was synthesized using the Maxima First Strand cDNA Synthesis Kit (Thermo Fisher Scientific, catalog no. K1672) per the manufacturer’s protocol. RT-qPCR was performed in 10-μl reactions using SYBR Green (Genesee Scientific, catalog no.17-505B) in a QuantStudio 6 Flex thermocycler (Applied Biosystems). Thermocycler conditions were as follows: 95°C for 10 min, followed by 40 cycles of 95°C for 15 s and 60°C for 60 s. Primers for all murine genes of interest were purchased (IDT, USA): *beta-actin–Actb* (reverse: CTCTTTGATGTCACGCACGATTTC and forward: GTGGCCGCTCTAGGCACCAA), *Glut1* (forward: GCTTCTCCAACTGGACCTCAAAC and reverse: ACGAGGAGCACCGTGAAGATGA), and *Hif1a* (reverse: CCATCAGAAGGACTTGCTGGCT and rorward: CCTGCACTGAATCAAGAGGTTGC) (*16*). Relative expression was determined using the ∆∆*C*t method with *Actb* as the housekeeping gene.

### Bacterial growth assays

Growth assays were performed as previously described with minor modifications (*68*). Briefly, overnight cultures for each strain were diluted in fresh M9 medium formulated with 25 mM d-glucose to OD_600_ = 0.1. A 10 μl of diluted culture was added in duplicate to a 96-well plate and 190 μl of sterile-filtered solutions of 2-DG (Thermo Fisher Scientific, catalog no. L07338.03) and GSK2837808A (LDHi, Cayman chemicals, catalog no. 20626) were prepared with 25 mM d-glucose. The plates were incubated in a Synergy H1 microplate reader (BioTek) for 24 hours at 37°C without agitation, and OD_600_ measurements were recorded every 30 min. Data are reported as the mean of three independent experiments.

### Glucose uptake assay

Macrophage glucose uptake was measured with a glucose uptake cell-based assay kit (Cayman, catalog no. 600470) following the manufacturer’s instructions. Briefly, BMDM cells were seeded at 1 × 10^5^ cells per well in black 96-well plates (catalog no. 3603) in DMEM + 10% heat-inactivated FBS. Cells were allowed to adhere for 18 hours at 37°C and 5% CO_2_. Then, cells were infected in DMEM without glucose (Gibco, catalog no. A1443001) at an MOI of 20, and the plates were centrifuged at 1000 rpm for 5 min at room temperature. Thirty min before the infection was ended, 2-[*N*-(7-nitrobenz-2-oxa-1,3-diazol-4-yl) amino]-2-deoxy-d-glucose (Cayman Chemicals, catalog no. 11046) at a concentration of 100 μg/ml in glucose-free media was added at the end of treatment, and plates were centrifuged at 1000 rpm for 5 min at room temperature. The supernatant was removed, and the cells were washed 2× with a cell-based assay buffer (Cayman Chemicals, catalog no. 10009322). The fluorescence signal (excitation/emission = 485/535 nm) was immediately analyzed in a plate reader (Sinergy H1, Biotek).

### LDH activity assay

LDH activity was assessed using a Cytotox-One Assay kit (Promega, catalog no. G7890). Briefly, J774.A1 cells were seeded at 5 × 10^4^ cells per well in black 96-well plates (catalog no. 3603) in DMEM + 10% heat-inactivated FBS. Cells were allowed to adhere for 18 hours at 37°C and 5% CO_2_. Then, cells were infected in serum-free DMEM (Gibco catalog no. A1443001) at an MOI of 2 and a volume of 100 μl, and the plates were centrifuged at 1000 rpm for 5 min at room temperature. After the treatment period of 4 hours, 50 μl of the Cytotox-One reagent, freshly prepared by mixing the substrate with assay buffer, was added to each well. The plate was incubated at room temperature for 10 min in the dark, after which 25 μl of the Stop Solution was added to terminate the reaction. The fluorescence signal (excitation/emission = 460/590 nm) was immediately analyzed in a plate reader (Sinergy H1, Biotek). To determine the LDH activity, the fluorescence from treatment wells (experimental LDH release) was compared to spontaneous LDH release (untreated control wells) and maximum LDH release (wells treated with Lysis Solution). Intracellular LDH activity was performed by subtracting the signal from the maximum LDH release from spontaneous LDH release.

### Lactate measurement

BMDMs were seeded in six-well plates (Corning, catalog no. 353060) at 1.5 × 10^6^ cells per well and allowed to adhere for 18 hours at 37°C and 5% CO_2_. Cells were infected at an MOI of 20 as determined by OD_600_ in serum-free media. Plates were spun at 1000 rpm for 5 min to settle bacteria into cells and then cocultured for 4 hours at 37°C and 5% CO_2_. The cell culture supernatant was collected for detection of l-lactate by using the Glycolysis Cell-Based Assay Kit (Cayman, catalog no. 600450) according to the manufacturer’s instructions.

### Polysome analysis

Polysome analysis was performed as described in previous publications (*49*, *50*). Briefly, cells were treated with CHX (100 μg/ml) at 37°C for 10 min before harvest. Cells were washed with PBS containing CHX (100 μg/ml), harvested by scraping, and pelleted by centrifugation at 10,000 rpm for 5 min at 4°C. Pellets were stored at −80°C until analysis. Cells were lysed in polysome lysis buffer [20 mM tris-HCl (pH 7.4), 140 mM KCl, 5 mM MgCl_2_, 0.1% Triton X-100, and 10 mM dithiothreitol] and sheared by passaging through a 27-gauge needle five times. Lysates were centrifuged for 5 min at 2500 rcf at 4°C to pellet nuclei, followed by centrifugation for 10 min at 13,000 rcf at 4°C to pellet mitochondria. Protein concentration was determined using the Bradford assay (VWR), and equal amounts of protein were layered onto a 10 to 50% linear sucrose gradient. Gradients were centrifuged at 35,000 rpm for 2 hours at 4°C without brake and fractionated into 12 equal parts while continuously monitoring ultraviolet absorbance at 254 nm to assess RNA abundance across the gradient. RNA was extracted from equal volumes of each gradient fraction using TRIzol. Equal volumes of RNA from each fraction were reverse transcribed, and cDNA from fractions corresponding to monosomes (fractions 3 to 6) and polysomes (fractions 8 to 11) was pooled separately. The transcript abundance was quantified by RT-qPCR using absolute quantification based on standard curves. Translation efficiency was calculated by comparing the abundance of each transcript in polysomal versus monosomal pools, expressed as a percentage of the total transcript distribution across the gradient.

### Statistical analysis

Normal data distribution was verified through the Shapiro-Wilk normality test, and parametric tests were used only for normally distributed data. Relative values of protein bands from Western blot films were assessed by ImageJ software (https://imagej.net/ij/index.html) (*65*). Correlation analysis using Pearson’s *r* was conducted to explore the relationship between HIF-1α (relative intensity) and intracellular bacterial load (CFU/ml). All statistical analyses, including Pearson’s correlation coefficient calculation, were conducted using GraphPad Prism 10 (GraphPad Software, Inc., CA, US). Significant differences are *P < 0.05, **P < 0.01, ***P < 0.005, and ****P < 0.001.
