# Supplementary material for: Yersiniabactin-producing adherent-invasive Escherichia coli exploit host glycolysis to drive macrophage HIF-1α stabilization
Source: Sci Adv. 2026 Jul 23;12(30):eaeb7972. doi: 10.1126/sciadv.aeb7972 (PMC13394464; doi:10.1126/sciadv.aeb7972)
Supplement: Supplementary file 1 — Figs. S1 and S2 [file sciadv.aeb7972_sm.pdf]

Science Advances
AAAS

Supplementary Materials for

Yersiniabactin-producing adherent-invasive *Escherichia coli* exploit host glycolysis to drive macrophage HIF-1α stabilization

Marlus S. Pedrosa *et al.*

Corresponding author: Janelle C. Arthur, janelle_arthur@med.unc.edu

Sci. Adv. **12**, eaeb7972 (2026)
DOI: 10.1126/sciadv.aeb7972

This PDF file includes:

Figs. S1 and S2

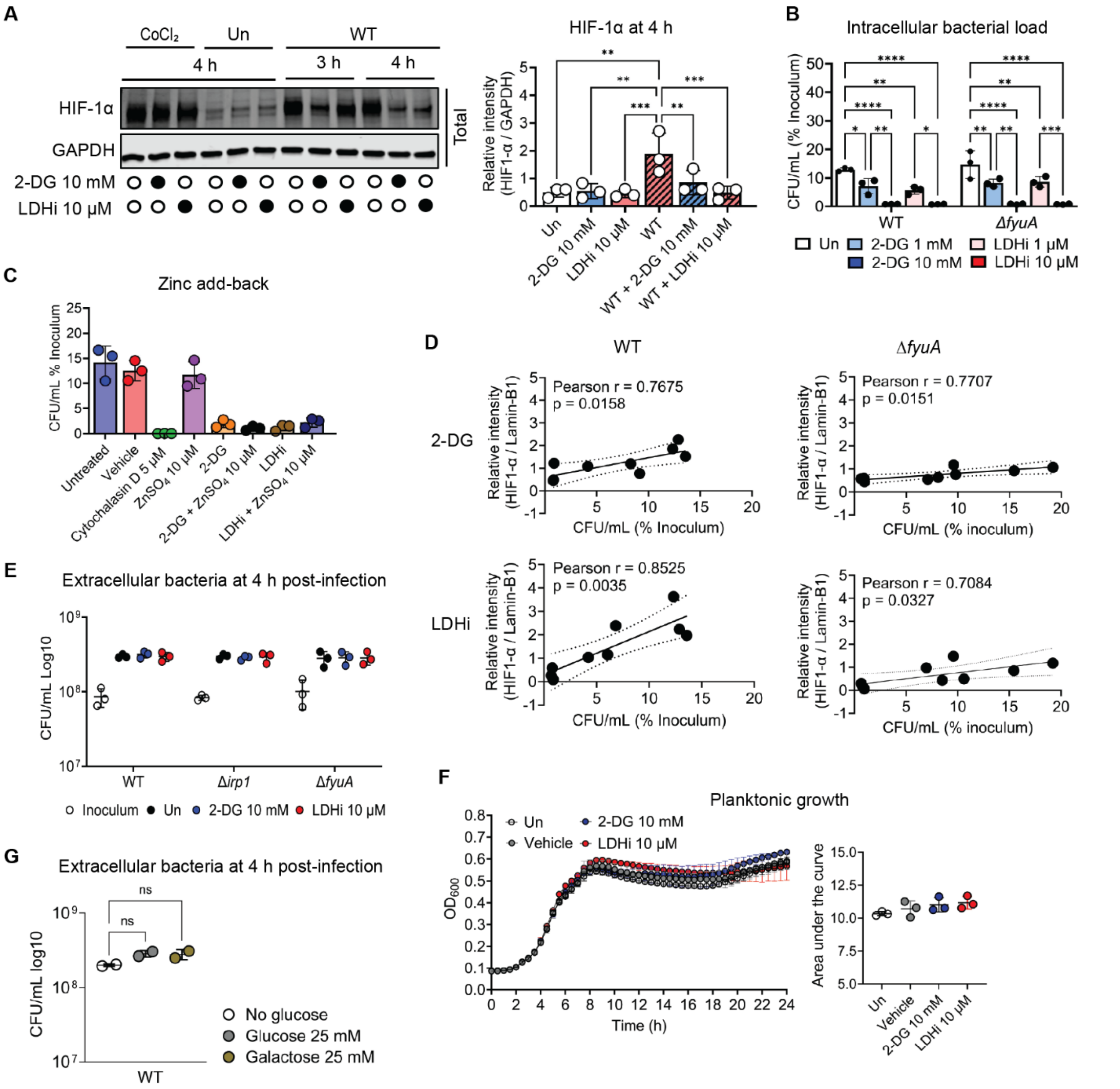

Fig. S1.

Glycolysis-dependent HIF-1α stabilization correlates with macrophage AIEC survival, independent of direct effect on AIEC growth. (A) BMDMs were treated with 2-DG (10 mM) or LDHi (10 µM) and infected with Ybt-producing WT for 3 and 4 h. Total cell lysates were used to quantify HIF-1α by western blot, with GAPDH as the loading control (left). The circles below indicate the presence (●) or absence (○) of the respective treatments indicated in the blots. HIF-1α relative intensity at 4 h was quantified using ImageJ and normalized to GAPDH (right). **(B)** J774.A1 macrophages were treated with 2-DG or LDHi and infected as in Figures 3D and 3E. Bacterial internalization after 4 h was quantified by gentamicin protection assay, presented as a percentage of inoculum, mean ± SD, n = 3. Statistical significance was assessed using one-way ANOVA with Tukey's post-hoc test. **(C)** BMDMs were treated with zinc ($ZnSO_4$; 10 µM) in the presence or absence of 2-DG or LDHi and infected with Ybt-producing WT. Bacterial

internalization after 4 h was quantified as above, mean ± SD, n = 3. **(D)** Pearson's r correlation coefficients were calculated between normalized HIF-1α relative intensity (Figures 3D and 3E) and intracellular CFU/mL (S1B). **(E)** J774.A1 macrophages were infected with WT, Δ*irp1,* and Δ*fyuA* NC101 for 4 h. The supernatant was collected, and extracellular bacteria were quantified by CFU/mL. **(F)** Growth curves of NC101 WT in M9 minimal media with 2-DG (10 mM) and LDH 10 µM (left). Area under the curve is shown (right). Data are presented as the mean ± SD from three independent experiments in duplicates. **(G)** BMDMs were infected with WT NC101 in glucose-free media and supplemented with D-glucose or D-galactose (25 mM). Extracellular bacteria were quantified from supernatants, mean ± SD from two independent experiments in duplicates.

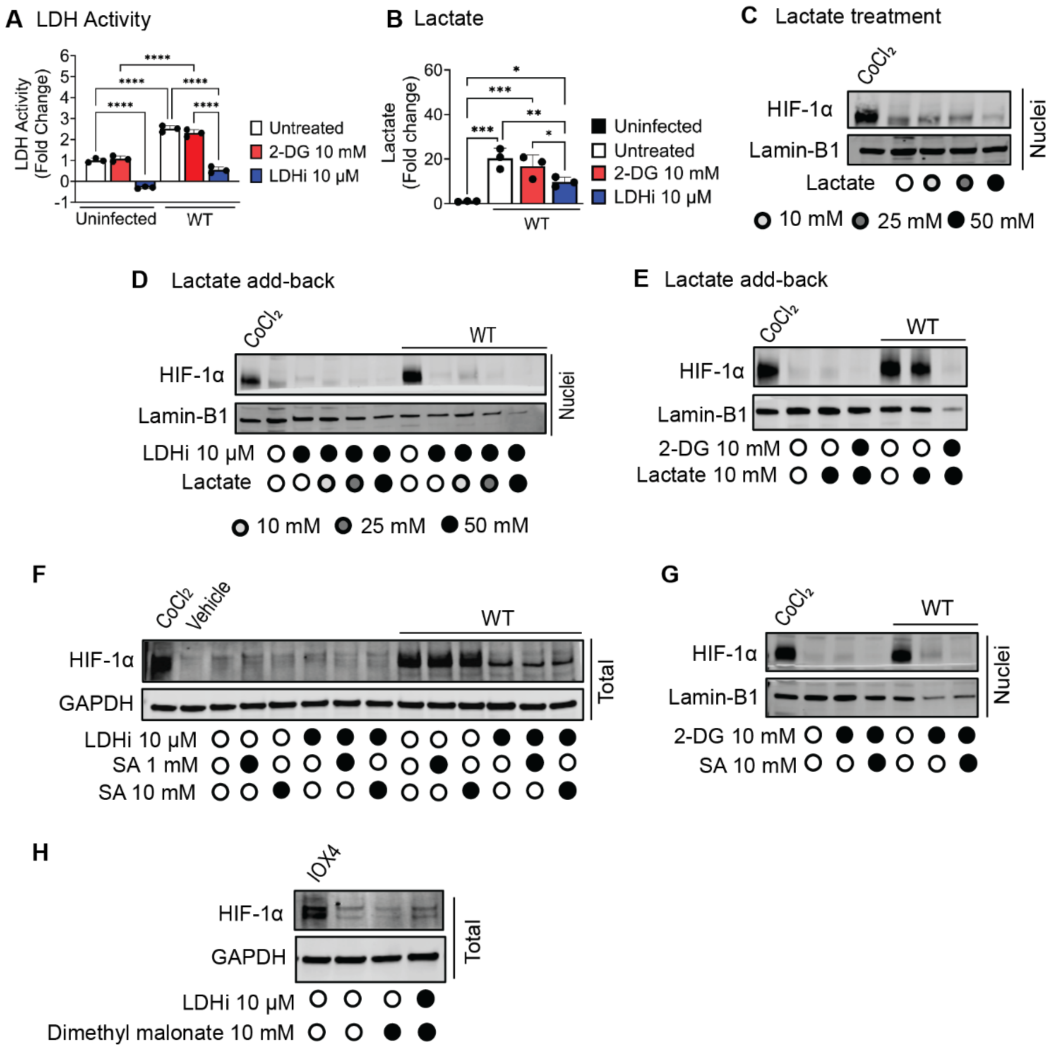

Fig. S2.

Lactate and succinate add-back do not restore HIF-1α stabilization by Ybt[+] AIEC. (A). LDH activity and **(B)** lactate levels of uninfected and WT NC101 infected J774.A1 cells treated with LDHi (10 µM) or 2-DG (10 mM). Data are presented as mean ± SD from three independent experiments performed in duplicates. Statistical significance was assessed using two-way ANOVA followed by Tukey's post-hoc test. **(C)** J774.A1 cells were treated with an equal ratio mixture of D and L-lactate (10 mM, 25 mM, and 50 mM) for 4 h. Nuclear extracts were used to quantify HIF-1α by western blot. **(D-E)** J774.A1 were treated with an equal ratio mixture of D and L-lactate (10 mM, 25 mM and 50 mM) and **(D)** LDHi (10 µM) or **(E)** 2-DG (10 mM) and infected with WT NC101 for 4 h. Nuclear extracts were used to quantify HIF-1α by western blot. **(F-G)** J774.A1 were treated with **(F)** succinate (1 and 10 mM) and LDHi (10 µM) or **(G)** succinate (10 mM) and 2-DG (10 mM) and infected with WT NC101 for 4 h. Nuclear extracts were used to quantify HIF-1α by western blot. **(H)** BMDMs were treated with dimethyl malonate (10 mM) or LDHi (10 µM). Total cell lysates were used to quantify HIF-1α by western blot. For all blots, Lamin-B1 and GAPDH were used as the loading control for nuclear fractions and total cell lysates, respectively. The circles below indicate the presence (●) or absence (○) of the

respective treatments indicated in the blots. All blots are representative of three independent experiments.
